# Nanowire-based integrated photonics for quantum information and quantum sensing

**DOI:** 10.1515/nanoph-2022-0652

**Published:** 2023-01-23

**Authors:** Jin Chang, Jun Gao, Iman Esmaeil Zadeh, Ali W. Elshaari, Val Zwiller

**Affiliations:** Kavli Institute of Nanoscience, Department of Quantum Nanoscience, Delft University of Technology, 2628CJ Delft, The Netherlands; Department of Applied Physics, Royal Institute of Technology, Albanova University Centre, Roslagstullsbacken 21, 106 91 Stockholm, Sweden; Department of Imaging Physics (ImPhys), Faculty of Applied Sciences, Delft University of Technology, 2628CJ Delft, The Netherlands

**Keywords:** epitaxial quantum dots, nanowires, photonics integrated circuits, quantum information processing, quantum sensing, superconducting nanowire single photon detector

## Abstract

At the core of quantum photonic information processing and sensing, two major building pillars are single-photon emitters and single-photon detectors. In this review, we systematically summarize the working theory, material platform, fabrication process, and game-changing applications enabled by state-of-the-art quantum dots in nanowire emitters and superconducting nanowire single-photon detectors. Such nanowire-based quantum hardware offers promising properties for modern quantum optics experiments. We highlight several burgeoning quantum photonics applications using nanowires and discuss development trends of integrated quantum photonics. Also, we propose quantum information processing and sensing experiments for the quantum optics community, and future interdisciplinary applications.

## 1 Introduction

Having the highest possible speed allowed by the laws of physics, photons are the fastest carrier to transmit information. The large bandwidth and bosonic behavior, allowing for photons to share (part of) a channel without a short circuit, have made photons the main choice for communication networks. Photonics is also a vital element in the toolbox of future quantum technologies. Conventionally, quantum optics experiments were carried out using tabletop equipment [1], [2], [3]. While such setups are flexible, accommodating components based on innovative technologies and material platforms, and have enabled the demonstration of a very impressive prototype to manipulate numerous photons [4], ultimately this cannot be scaled much further. Over the past 25 years, integrated photonics was established as a reliable, scalable, and cost-efficient alternative to bulk optics [5], [6], [7], [8] and offers exciting prospects for intense upscaling.

**Table 1:** Overview of the nanowire fabrication and materials choice for representative quantum emitters and detectors.

Materials	Quantum dots in nanowire single-photon emitters		Reference
	Fabrication method	Key performance	
InAs/GaAs	Solid-source MBE	Emission at 1300 nm	[77]
InAsP/InP	Chemical beam epitaxy	Emission 880–1550 nm	[34]
InAsP/InP	MOVPE	Light-extraction efficiency of 42%	[27]
InAsP/InP	Chemical beam epitaxy	High-fidelity entangled photon-pairs	[29]
InAsP/InP	Vapor–liquid-solid (VLS) epitaxy	Multi-photon event <1%	[78]
AlGaAs/GaAs	MBE	Background-free	[24]
GaAsP/GaP	Low-pressure MOVPE	Bright QDs grown on Si substrate	[79]
InGaN/GaN	Plasma-assisted MBE	Electrically driven QDs	[80]
InGaAs/GaAs	MOCVD	Strain-engineered telecom wavelength DQs	[81]
AlGaN/GaN	MOCVD	Room-temperature operation with $g_{0}^{\left(2\right)}=0.13$	[82]
**Materials**	**Superconducting nanowire single-photon detectons**		**Reference**
	**Fabrication method**	**Key performance**	
NbN	Molecular-beam epitaxy (MBE)	Working on AlN *χ*^(2)^ circuits	[83]
NbN	Atomic layer deposited (ALD)	Working till 2006 nm	[84]
NbTiN	Magnetron co-sputtering	>99% efficiency	[61]
NbTiN	Magnetron co-sputtering	7.7 ps timing jitter	[85]
WSi	Magnetron co-sputtering	93% efficiency	[86]
MoSi	Magnetron co-sputtering	>98% efficiency	[87]
NbRe	Magnetron sputtering	Visible-infrared detection	[88]
TaN	Magnetron sputtering	Large-area X-ray detection	[89]
NbN	Magnetron sputtering	>98% efficiency	[90]
MgB_2_	Hybrid physical chemical vapor deposition	130 ps relaxation time	[91]

Quantum photonics has taken a similar progress path as the broader field of optics, i.e. starting with bulk free-space equipment and miniaturization via integration. Integrated quantum photonics (IQP) has already gone a long way and milestone theoretical and experimental works on IQP have already been reported in different platforms [9], [10], [11], [12], [13], [14]. However, many challenges still prevent scaling such platforms. As we argue in this paper, the most formidable challenge ahead for IQP is compatibility issues: performances of individual quantum integrated photonics components, i.e. emitters, photonic circuits, and detectors, have excelled in the past two decades [15], [16], [17], but these elements are either incompatible or their integration comes at the cost of significant performance penalties. Hybrid integration is a method in which individual elements are created in their compatible platform and environment, and are then transferred to a host substrate. These approaches have gained significant attention, and several independent works have demonstrated the viability and potential of these techniques.

Nanowire-based integrated photonics is an important member of the hybrid integration class and the focus of this paper. We organize this review in the following structure: After a general introduction to integrated quantum photonics in Section 1, we discuss nanowire-based emitters in Section 2, integrated nanowire detectors in Section 3 and then review the material and fabrication methodologies in Section 4. Section 5 summarizes promising integrated quantum photonics applications enabled by nanowire technology and Section 6 is dedicated to prospects and promising future applications of nanowire-based, quantum-enhanced photonic technology followed by a conclusion in Section 7.

## 2 Quantum emitters

Numerous quantum information technologies rely heavily on non-classical light. In particular, several quantum-secured communication and quantum computing techniques need light sources that can generate single photons, entangled photon pairs, or cluster states as a necessary resource. For the creation of non-classical light, sources based on solid-state nanoscale emitters have emerged as a high-quality and potentially scalable option in the last few years [18]. A variety of solid-state emitters are utilized to generate non-classical light, including carbon nanotubes [19], semiconductor quantum dots (QDs) [20], and 2D materials [21]. Due to their high emission rate [22], narrow emission line width [23], record low multi-photon emission probability [24], and high indistinguishability [25], QDs are often used in advanced applications for the demonstration of quantum advantage [26]. In as-grown planar quantum dot samples, the light extraction efficiency is very limited due to the large refractive index contrast of the host material. Recently, there has been a rapid development in producing single photon sources with superior light extraction efficiency [27], and small fine structure splitting [28, 29], through embedding QDs in a photonic nanowire. In these nanowires, the size and placement of the QDs are well controlled, resulting in excellent spectral purity [30]. For an in-depth review of nanowire-based sources of non-classical light sources, we refer the reader to reference [31].

There are two main bottom–up methods to fabricate nanowire-based QDs [32]: Selective-area epitaxy, where the nanowire is grown on a patterned substrate, and vapor–solid–liquid epitaxy, where the metal catalyst is used to grow the nanowire. Such techniques result in ultra-bright and clean emission from nanowire quantum dots approaching the Fourier-transform limit [30] with a Gaussian mode emission profile and near unity coupling to the guided optical mode, enabling high collection efficiency [33] and large operation bandwidth through controlling the growth conditions. As an example, the typical emission wavelength of InAsP quantum dots in InP nanowires is around 900 nm, by controlling the size of the quantum dot in the growth phase, which sets the confinement potential, the emission wavelength can be extended to the telecom range [34] while maintaining a Gaussian emission profile for efficient optical fiber coupling [35].

The uniqueness of the bottom–up growth method of nanowires mitigates a number of possible processes that may cause linewidth broadening. For example, each device can contain a single or several quantum dots in a highly controlled process [36]. Additionally, dot nucleation can happen without the development of a wetting layer, and the sidewalls of the photonic nanowire are epitaxially formed crystal planes rather than etched using dry or wet etching methods. Although multi-emitter circuits have been realized [37], one standing challenge is to tune all the quantum sources to the same operating wavelength for achieving high two-photon interference visibility. Piezoelectric and thermal tuning have been realized experimentally to control the QDs emission wavelength [37, 38], and an electrostatic approach through Stark-shift has been recently theoretically proposed [39]. It is still an open question whether only wavelength tuning is sufficient to reach high photon indistinguishability, without the need for Purcell enhancement. On short time scales, the indistinguishability of photons is governed by *T*_2_/2(*T*_1_), where *T*_1_ is the emitter lifetime and *T*_2_ is the coherence time. Also, $1/T_{2}=1/2T_{1}+T_{2}^{*}$ , where $T_{2}^{*}$ is defined as the pure dephasing time deviating from the natural line width [40]. Recently, by using a highly optimized growth process, the line widths of nanowires QDs emission spectra were shown to be only 2 times of the Fourier transform limit for above-band excitation [30]. Although further measurements are needed to characterize indistinguishability through two-photon interference visibility, this important result further demonstrates the great potential of nanowire quantum dots for quantum information processing.

In addition to the attractive single photon emission properties, high-fidelity spin-qubit initialization using optical pumping was recently realized in nanowire quantum dots. Such solid-state qubits can be potentially used for quantum repeaters to generate entanglement between flying and anchored qubits [41]. Additionally, nanowire quantum dots can produce polarization-entangled photon pairs through the biexciton-exciton cascade [28, 29]. The process makes use of the Pauli exclusion principle in the quantum dot’s s-shell. A completely filled s-shell leads to a zero-spin bound biexciton state, and two cascaded photons are then emitted with zero total angular momentum. Since the photon-pair state cannot be factorized (into a product state of each individual photon’s polarization state), the polarization state of the photon pair is a maximally entangled Bell state. Time-bin entanglement was also realized with nanowire quantum dots [42], which may be more suitable for long-distance communication in optical fibers. Additionally, the growth of nanowire quantum dots can be tailored to tune the emission wavelength (e.g. by strain engineering) to interface with atomic memories, which is of paramount importance for quantum memories and quantum repeaters [43], [44], [45], [46].

**Figure 1:**
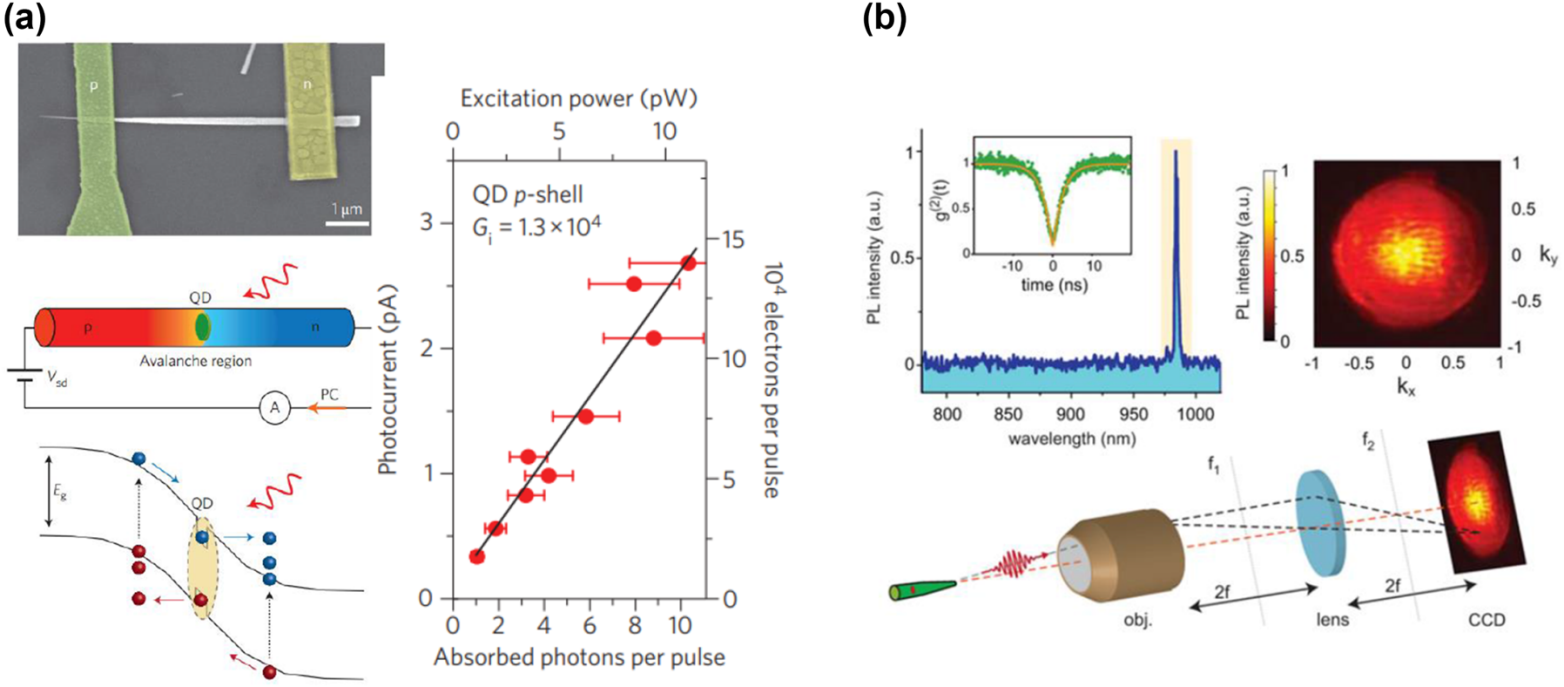
Nanowires, due to their unique geometry, offer the possibility to both detect and emit light. (a) An embedded QD in a nanowire is contacted and used as an efficient light detector [51]. (b) Nanowires with engineered geometry can efficiently guide light emitted by QDs and beam-shape the emission into a near-perfect Gaussian, compatible with standard single-mode optical fibers [35]. Reprinted with permission from Bulgarini, et al. [35], Copyright 2014 American Chemical Society.

Besides promising emission properties, QDs can also be used for single-photon detection [47]. In principle, QD-based detectors, when designed appropriately, can be sensitive to a wide range of wavelengths, extending to the mid-infrared [48]. Recently, semiconductor nanowire-based light detectors have shown very promising detection performances [49]. Nanowires, when utilized as in-plane light detectors (integrated with the plane of photonic circuits), have the added advantage of being compatible with integrated photonics [50, 51]. An example of such a detector is shown in Figure 1(a). Semiconductor nanowire detectors still have a long way to go to match the performance metrics of other on-chip single-photon detection technologies (such as superconducting nanowires, which will be covered in Section 3), but thanks to their potential room temperature operation condition and compatibility with monolithic integration techniques, they hold great promises for future integrated quantum photonics.

**Figure 2:**
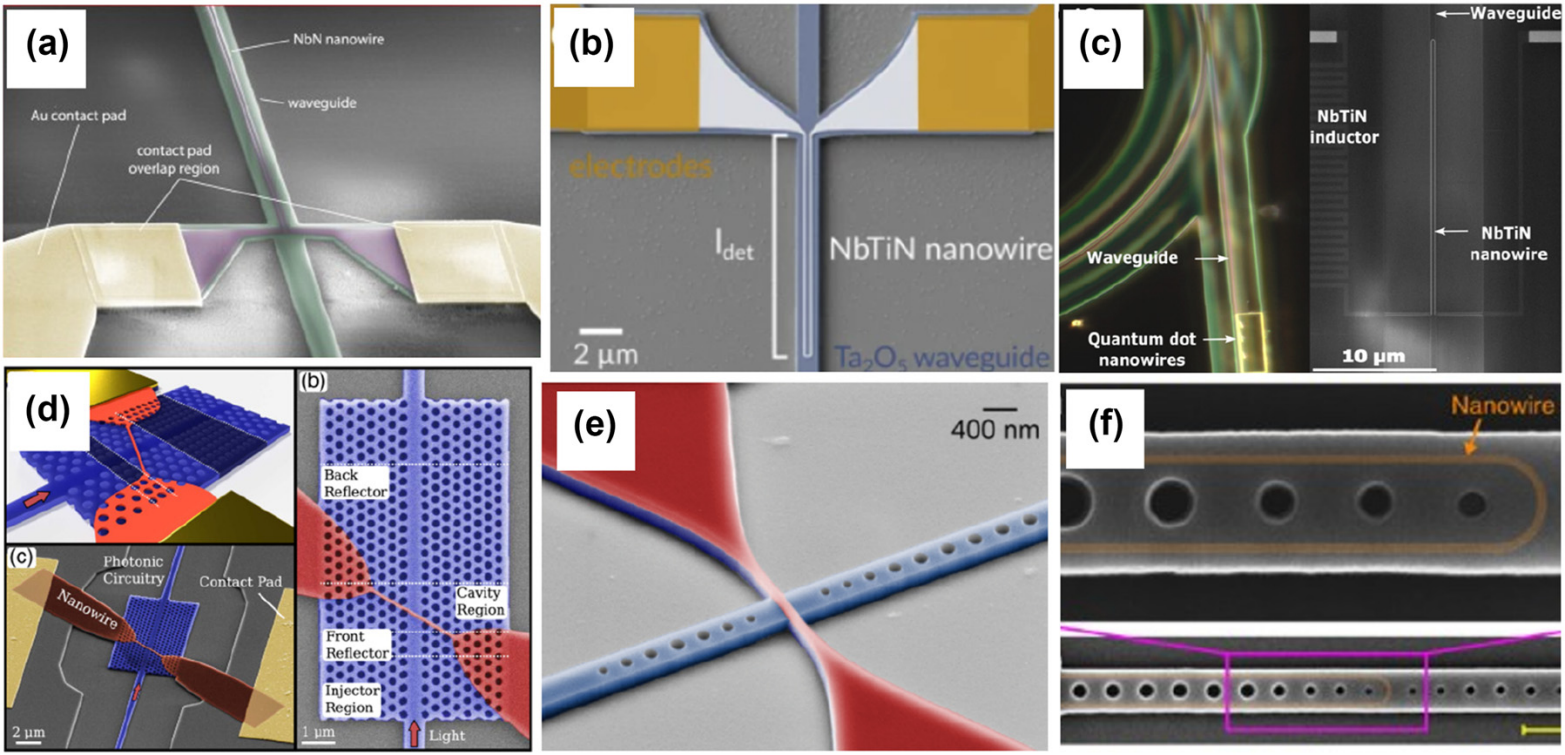
Superconducting nanowire on-chip integration with different optical structures: (a) SNSPDs embedded in silicon nitride nanophotonic circuits with internal quantum efficiencies close to unity at 1550 nm wavelength [55]; (b) NbTiN superconducting nanowire integrated with Ta_2_O_5_ waveguide achieving 75% on-chip detection efficiency at 1550 nm [56]; (c) optical microscope picture of a part of the integrated photonic circuit including quantum dots nanowires, a waveguide, and a ring resonator. Reprinted with permission from Gourgues et al. [57] © The Optical Society. (d) short NbN superconducting nanowire integrated into two-dimensional double heterostructure photonic crystal cavity with recovery times of 480 ps. Reprinted with permission from Munzberg et al. [58] © The Optical Society. (e) nanobeam cavity-integrated NbN superconducting nanowire single-photon detectors with sub-nanosecond decay and recovery times [59], Reprinted with permission from Vetter et al., Copyright 2016 American Chemical Society, and (f) U-shaped NbTiN nanowires atop silicon-on-insulator waveguides are embedded in asymmetric nanobeam cavities with a near unity on-chip quantum efficiency for 1545 nm [60].

## 3 Integrated nanowire single-photon detectors

The inception of superconducting nanowires for single-photon detection dates back to 2001 [52]. Since then, the superconducting nanowire single-photon detectors (SNSPDs) field has witnessed great progress and improvement, mostly with standard fiber-coupled devices [17]. The working principle of on-chip integrated SNSPDs is the same as conventional fiber-coupled detectors [53], typically superconducting nanowires are DC-current biased below their critical current and temperature *T*_*c*_ and when a photon is absorbed, cooper pairs are broken thus quasi-particles (and/or vortices) are created. This leads to the formation of a normal-conducting region in the wire, redirecting the current toward the readout electronics. After a certain recovery time, the superconducting nanowires return to their superconducting state, and the dynamics of this process depend on the kinetic inductance of the device and the readout circuitry.

However, fiber-coupled SNSPDs are not favorable for scaling up detector numbers and lowering the cost per detection channel. For many quantum optics experiments, detectors are usually separately placed in a closed-cycle cryostat, which increases the total cost of targeted applications [4, 26, 54]. Integrating multiple SNSPDs on-chip would enable large-scale on-chip quantum optics experiments with a more compact chip-scale design and fabrication.

In recent years, great efforts have been put into the integration of large numbers of single-photon detectors on-chip. Unlike traditional fiber-coupled SNSPDs [61], in order to achieve high detection efficiency, SNSPDs are either integrated into photonic waveguides with traveling wave geometry [62, 63], or placed in planar photonic crystal cavities [58, 59]. SNSPDs are typically placed atop optical waveguides and the traveling light field is absorbed by sufficient long superconducting nanowires. This requires depositing superconducting thin films on top of the waveguide layer and then performing electron beam lithography followed by etching steps; an alternative approach is to embed the superconducting nanowires into optical waveguides. As shown in [57], SNSPDs can first be fabricated and tested, and then the optical waveguides can be deterministically formed (deposited and patterned) to integrate with pre-selected SNSPDs.

When designing on-chip SNSPDs, the main concerns include choosing materials, and fabrication routine as described in the following Section 4. Due to the small footprint and lower kinetic inductance of the on-chip SNSPDs, these detectors can exhibit faster (sub-nanosecond [58]) recovery time and a lower dark count rate compared to fiber-coupled devices. Also, since the total length of integrated nanowires is significantly shorter, the probability of introducing imperfections is reduced thus a higher yield can be expected. In the future, we expect large numbers of SNSPDs or SNSPD cameras to be integrated into quantum photonic chips for achieving more sophisticated tasks. For a more detailed review of the on-chip integration of SNSPDs with different types of waveguides and their performance, interested researchers are referred to [64]. In the future, besides deploying large numbers of detectors on-chip, how to read out the detection signals of thousands of detection channels remains as an outstanding challenge. In Section 6.1, we propose a hybrid integration architecture, which utilizes both the advantage of photonics and electronics technology to overcome the signal read-out challenge. Several representative integrated SNSPDs works are shown in Figure 2.

## 4 Material and fabrication for quantum emitters and detectors

**Figure 3:**
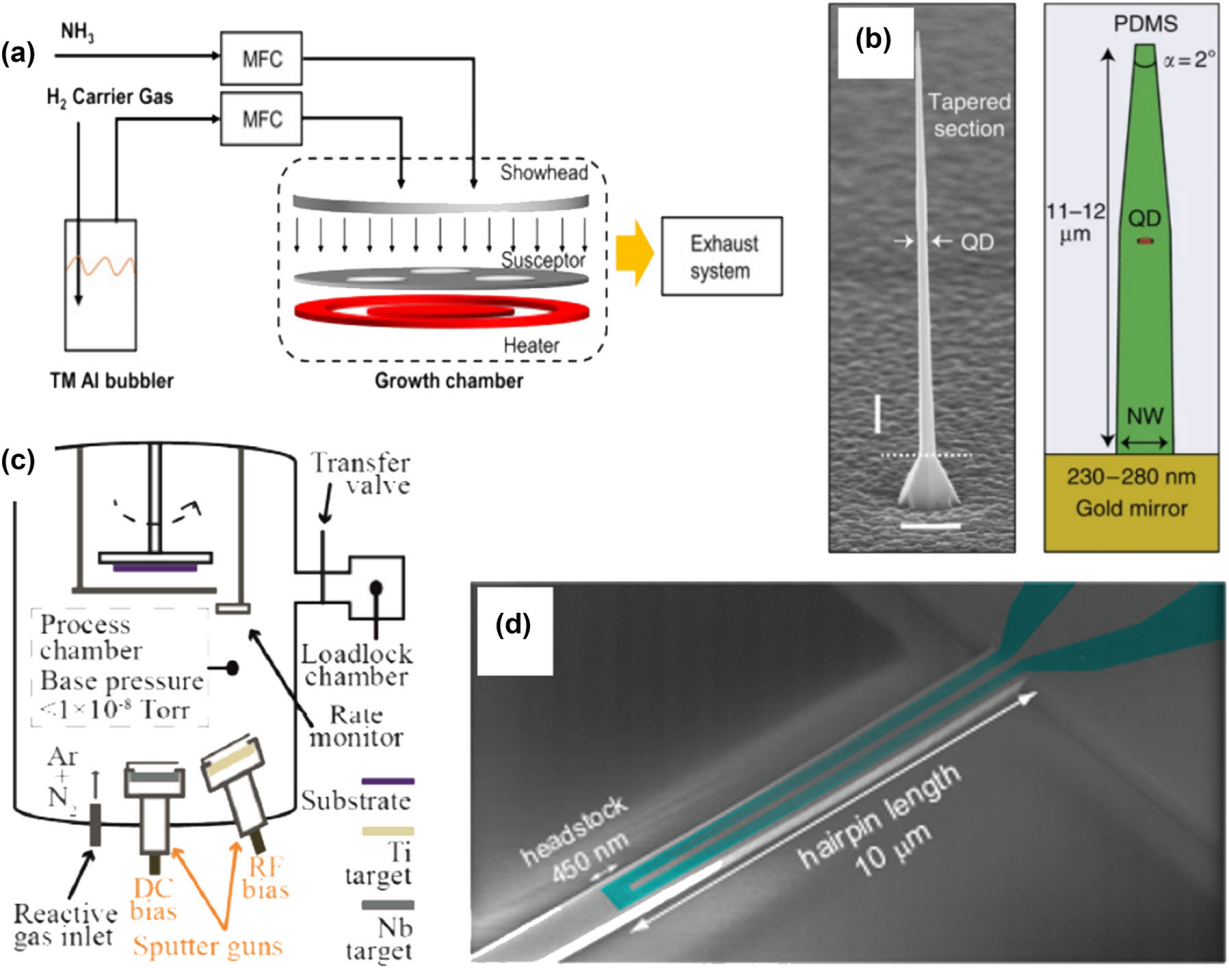
Representative fabrication technologies and device images for quantum emitters and quantum detectors: (a) the schematic of an MOVPE system for growing QDs in nanowire single-photon emitters [92]; (b) left is an SEM image of an InAsP quantum dot embedded in tapered InP nanowire waveguide; right image is tailored nanowire geometry embedded in polymer with bottom gold mirror [27]. We acknowledge the authors M. E. Reimer, et al. and Springer Nature publishing group for reusing the figure; (c) schematics of a magnetron co-sputtering system for depositing superconducting thin films. Reprinted with permission from Zichi et al. [68] © The Optical Society, and (d) false-color SEM image of a MoSi hairpin SNSPD on SOI waveguide [93]. Reprinted with permission from Li et al. [93] © The Optical Society.

In this section, we summarize representative material candidates and the general fabrication process for integrated QDs emitters and superconducting nanowire single-photon detectors. Also, we highlighted different hybrid nanowire device integration approaches, and focus on the integration methods and their challenges and advantages in terms of selectivity and scalability. An overview of representative nanowire-based quantum emitters and detectors' fabrication methods is shown in Figure 3, and their materials choices as well as key performance are summarized in Table 1.

### 4.1 Epitaxial growth of nanowire quantum dot

Semiconductor quantum dots have been systematically studied as single-photon sources in various quantum optics applications. They are typically fabricated with top–down or bottom–up approaches. Epitaxial methods, for example, molecular-beam epitaxy (MBE) or Metalorganic vapor-phase epitaxy (MOVPE) [65, 66] are frequently used for growing nanowire QDs. During the epitaxial process, short segments of smaller-band-gap semiconductors are embedded in a larger-band-gap semiconductor. Taking the InAsP QD in InP nanowire as an example [27]: InP nanowires were first grown on InP substrate in an MOVPE reactor with Au particles as catalyst and trimethyl-indium plus phosphine as precursors. Afterward, an InAsP quantum dot was incorporated by introducing As in the reactor using an arsine flux. Afterward, the chamber temperature was raised to favor radial versus axial growth, thus forming the InP shells. By controlling growth time and temperature, the nanowire geometry is shaped with an optimum nanowire diameter and tapering angle towards the tip. With the tapered waveguide structure, such QDs have high photon extraction efficiency. Each QDs nanowire can be individually tested and transferred following optical measurements to select the best quantum dots on photonic chips for more advanced quantum optics measurements and integration in complex architectures. For more detailed material and fabrication methods regarding nanowire QDs, we refer to [67].

### 4.2 Superconducting film deposition and detector integration

For superconducting nanowire single-photon detectors, the most commonly used film deposition technology is magnetron sputtering. It is a physical vapor deposition (PVD) process, where magnetically confined plasma is created near the surface of a target material (e.g. titanium or niobium). Positively charged energetic ions from the plasma collide with the negatively charged target material, and atoms from the target are “sputtered”, and then deposit onto the substrate [68]. A single target made of alloys or multiples targets each containing an elementary material can be used for thin superconducting film deposition. Afterward, with one-step electron beam lithography followed by reactive ion etching, the nanowire pattern is created on different substrates previously chosen for sputtering. An alternate approach is to fabricate and pre-test the superconducting nanowires, then deposit waveguide materials on top and selectively etch them to cover the detectors with waveguides [57]. Due to the shorter lengths of integrated superconducting nanowires and thus fewer imperfections, the yield, and recovery time of on-chip detectors can be significantly improved.

### 4.3 Hybrid integration of quantum emitters and detectors

Given the variety of the needed building blocks for single photon generation to manipulation and detection, a monolithic material platform will not be sufficient to realize complex photonic systems. Recently, there has been a rapid development in hybrid photonic integration approaches. Nanowire quantum emitters pioneered the field of hybrid quantum photonic integration [7]. The geometry of nanowires not only allows for efficient light extraction [27, 35], single-photon detection [51], and electroluminescence [69] 1, but also enables their transfer from the growth chip to other materials and platforms, such as piezoelectric crystals and silicon-based photonic circuits [37, 38, 46, 57, 70, 71]. Two methods of transfer can be used: nonselective through dispersing the nanowires randomly on a target sample and then building the photo-electronic circuitry that incorporates the nanowires using lithographic techniques [72], or selective site-controlled technique using pick and place transfer [38]. In the latter, a typical setup consists of a tungsten needle with a 100 nm tip diameter mounted on a high-precision XYZ stage. The needle can be controlled to adhere to a specific nanowire on the growth chip through van der Waals forces. Then, the tip is used to break the nanowire from the growth chip and transfer it to the target chip with a marker field for further alignment in subsequent fabrication steps. In addition to the pick-and-place technique, transfer printing approaches were also developed [73, 74]. Using a high-precision positioning system, a rubber stamp composed of polydimethylsiloxane (PDMS) can be used to transfer suspended structures from a growth chip to a target. While pick-and-place and transfer printing techniques offer high selectivity of the target quantum emitter in terms of emission line width and wavelength, the scalability is limited, as each quantum emitter has to be mechanically transferred to the target chip. Another promising approach, which over larger scalability, at the expense of selectivity, is the wafer bonding approach. III-V epitaxially produced QD sources have been successfully bonded to silicon nitride photonic chips using this technology [75]. Then using mechanical grinding, chemical mechanical polishing, or chemical etching, the sacrificial layer is removed once the bonding has occurred to reveal the photonic circuit layer. For more details about hybrid integration, we refer the readers to [7, 76]. Finally, hybrid integration was not only limited to quantum emitters, recently, SNSPD hybrid integration was also realized with 100% yield. In the future, interfaces between SNSPDs and external electronic will be necessary for the coupling of SNSPDs with intricate, dynamically reconfigurable photonic structures for active feedback operations.

## 5 Emerging quantum photonic technology and outlooks

In this section, we highlight a non-exhaustive number of established and emerging quantum optics applications enabled by (partially) integrating quantum emitters, waveguides, and detectors on-chip. We also present perspectives on future quantum technologies with their benchmarks and targets using integrated quantum photonic technology.

### 5.1 Photonic boson sampling

**Figure 4:**
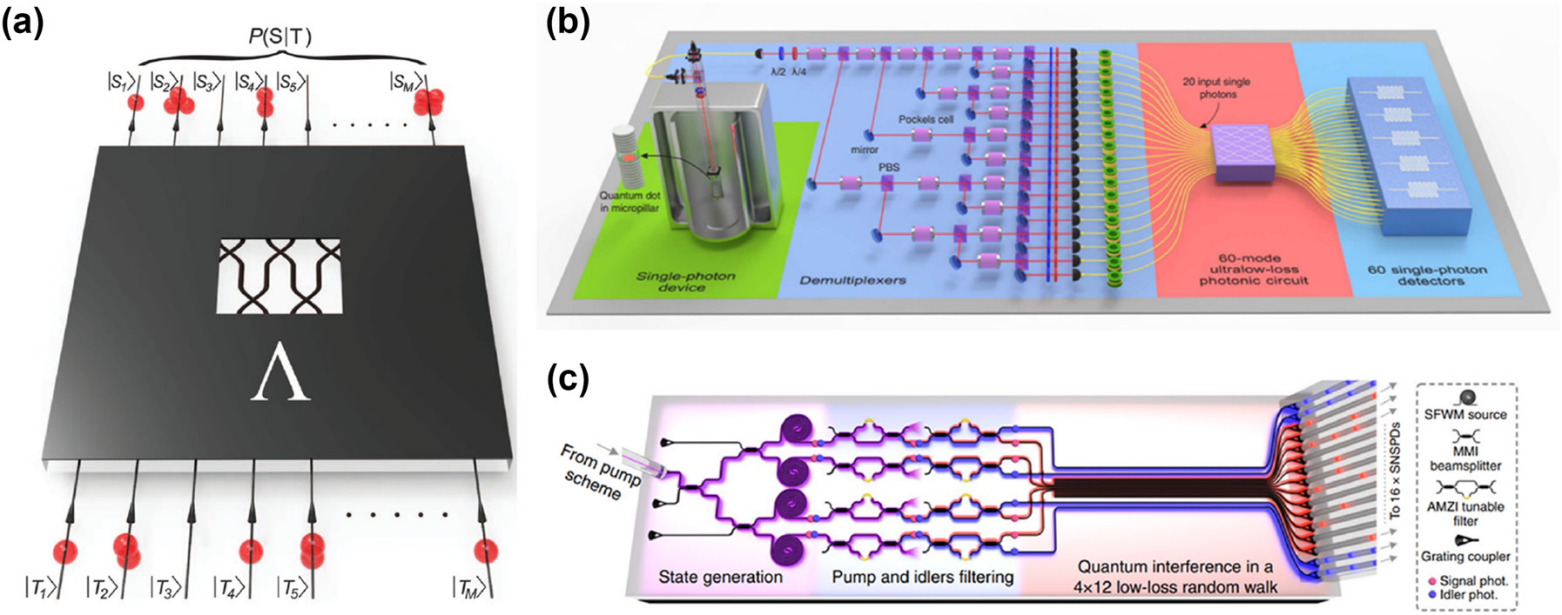
Boson sampling experiments using Fock states and Gaussian states as the input: (a) schematic of a boson sampling machine: identical bosons are prepared and interfere in a linear optical network, with the output distribution efficiently sampled from the linear network [94]. (b) Indistinguishable photons are generated by a semiconductor quantum dot and de-multiplexed in different spatial modes. The photons interfere in an ultra-low loss bulk crystal and are detected by nanowire single-photon detectors [95]. Reprinted figure with permission from Wang et al. [95]. Copyright 2019 by the American Physical Society. (c) On-chip generation of non-classical Gaussian light. The silicon photonic chip also integrates filters and a passive linear network to perform Gaussian boson sampling [96].

Boson sampling, first proposed by Scott Aaronson and Alex Arkhipov [97], is a computational task aiming to demonstrate quantum advantage with an intermediate-scale quantum device (Figure 4). The central idea of the task is to sample the output distribution of indistinguishable bosons interfering in a linear network, as schematically shown in Figure 4(a). With the increase of photon numbers, the task becomes intractable using the classical computation approach due to the intrinsic hardness of calculating the matrix permanent, and thus is considered an excellent candidate to demonstrate quantum computational advantage [98]. To this end, the photonic system is one of the most suitable platforms, as the key elements (quantum emitters, linear state evolution, and single-photon detectors) are widely available with the current technology as described in the previous Sections 2 and 3.

As the early demonstrations of boson sampling, several research groups have chosen to use photon pairs generated from spontaneous parametric down-conversion process and silica photonic chips [94, 99], [100], [101]. To overcome the low generation rate issue from probabilistic photon sources, some research groups chose semiconductor quantum dot emitter as the input states [26, 102], thus the photon number was dramatically increased from the initial 3 to 20 photons with a state space dimension up to 10^14^ [95]. The experimental setup shown in Figure 4(b) represents the largest scale of boson sampling using quantum dot emitter and SNSPD detection. In the meanwhile, a variant of boson sampling was proposed to use a single mode squeezed vacuum state as the input state instead of the Fock state, called Gaussian boson sampling [103, 104]. Unlike the original boson sampling protocol, where photon numbers are conserved, Gaussian boson sampling offers a boost in the photon number since the source could emit random numbers of photon pairs. Gaussian boson sampling has been experimentally demonstrated using both ultra-low loss bulk optics [4, 54] and silicon photonics platform [96]. Figure 4(c) demonstrates the design of the integrated photonics circuit, unlike the bulk optics setup, the chip has a rather small footprint and could be extended to a large-scale device. Nonlinear effects in silicon can naturally generate photon pairs via spontaneous four-wave mixing in either spiral waveguides [105] or ring resonators [106, 107]. It is still an open question whether the current technology is capable of scaling boson sampling to arbitrarily large dimensions while maintaining the quantum advantage. Also, such ultra-large-scale experiments require huge numbers of single-photon detectors and the corresponding coincidence detection systems, e.g. distributed SNSPDs with cryogenics and control electronics, making them far from cost-effective [108]. As a result, integrated photonics technology is commonly believed to be a promising approach to reaching scalable boson sampling [14]. In addition, the optical modes of Gaussian boson sampling can be mapped to vibrational normal modes to solve the vibronic spectrum of a molecule [109]. Gaussian boson sampling also holds the potential to solve graph theory problems [110, 111] and molecular docking for pharmaceutical drug design [112]. The on-chip photonic circuits potentially possess higher stability (e.g. less phase drift and frequency drift), better isolation from the environment, and low power consumption to be programmable [113] to solve the challenging applications we mentioned above. With the advances in integrated photonics technology, nowadays larger modular linear optical circuits [114], bright quantum emitters [27], and controlled integration of detectors [57] are more widely available, thus a scalable integrated boson sampler for specific practical problems is the next step to be achieved.

### 5.2 Quantum walks

**Figure 5:**
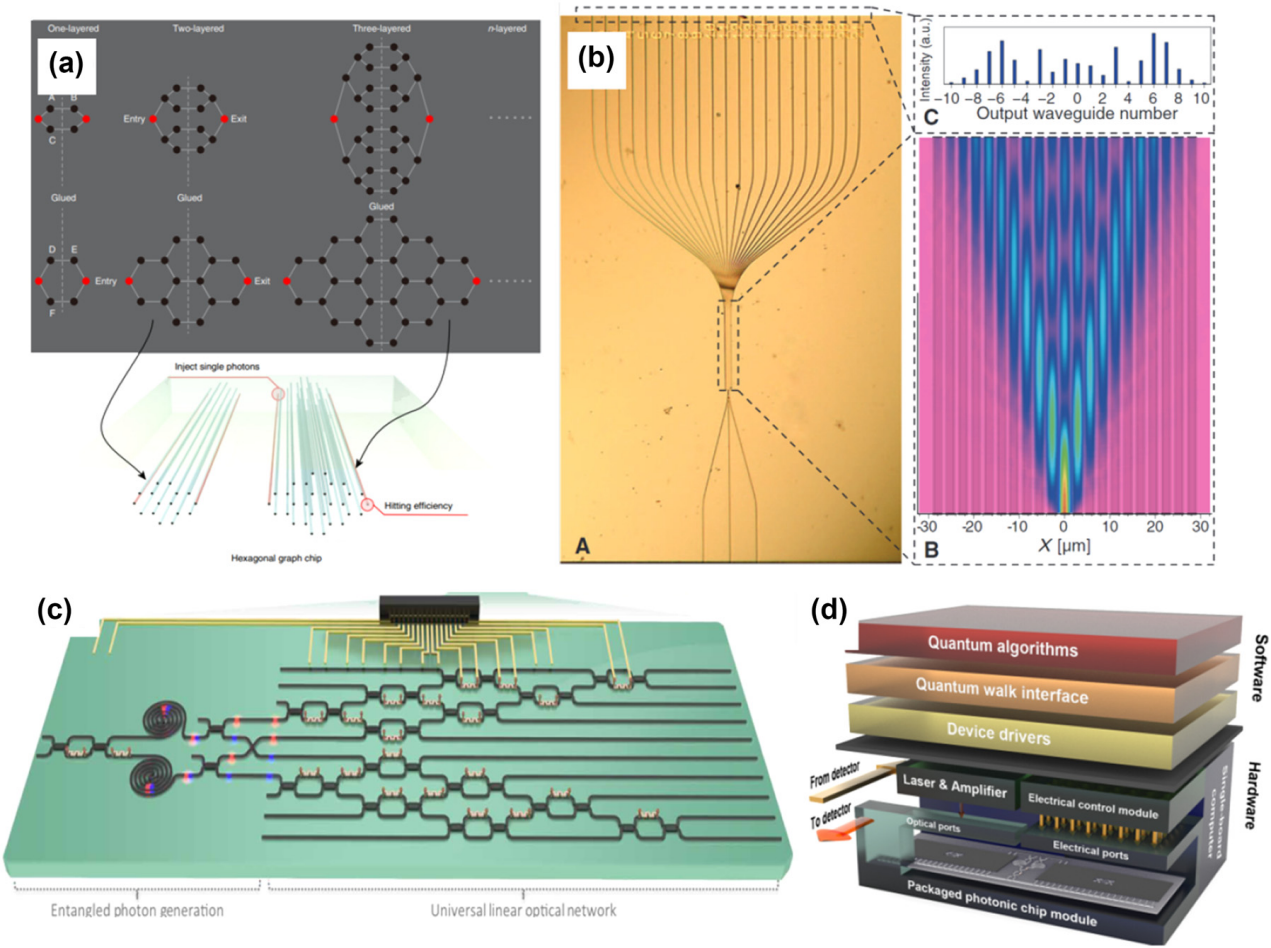
Representative quantum walk works (a) top: theoretical graphs of quantum fast-hitting using two-dimensional hexagonal structure; bottom: implementation of a quantum walk with a glued binary tree structure on photonic chips using femtosecond laser-written waveguide arrays [115]; (b) left: a continuously coupled waveguide array for realizing correlated photon quantum walks with 21-waveguide array; right: simulation and experiment output pattern of 810-nm laser light propagating through the waveguide array [116]; (c) graph-theoretic quantum algorithms on a silicon photonic quantum walk processor, including generating spatial-entangled photons and implementing universal five-dimensional unitary process [117], and (d) schematic of a large-scale full-programmable quantum walk system stack, where software stack compiles quantum algorithms into quantum walk settings and then operated by the hardware [118].

Quantum walks are the quantum counterparts of classical random walks first proposed in 1993 [119], where quantum superposition plays an extremely important role. Unlike a classical particle, a quantum particle can simultaneously propagate in different directions, and this unique behavior leads to the ballistic transport feature of the quantum walk. Normally, there are two different models of quantum walks, namely discrete quantum walks and continuous quantum walks. Depending on the tasks, quantum walks could provide either exponential (quantum fast-hitting) [115, 120] or polynomial (quantum search algorithm) [121, 122] speedup over classical algorithms [123], and could even implement universal quantum computation [124, 125]. The photon, which inherently exhibits the wave-particle duality, is naturally a good candidate as a “walker”. The quantum properties of photons including superposition, quantum interference, and entanglement can be employed to perform various quantum walk experiments. Early-stage quantum walk experiments are conducted by the bulk optics beam splitters [126] and fiber loops [127, 128] in the time domain. As a comparison, integrated circuits offer higher phase stability, thus further leading to a higher level of integration [116, 129, 130]. For example, Figure 5(a) illustrates the implementation of a quantum walk with a glued binary tree structure on photonic chips using femtosecond laser-written waveguide arrays, and Figure 5(b) demonstrates a continuously coupled waveguide array for realizing correlated photon quantum walks. Similarly, in Figure 5(c), an integrated photonic platform consisting of reconfigurable linear optical networks and controllable on-chip entangled photon pair sources, demonstrates the simulation of thousands of continuous-time quantum walk evolutions [117]. Most recently, a full-stack quantum walk processor based on an integrated photonic chip is used to demonstrate a series of quantum applications, from graph-theoretic applications to quantum simulations of topological phases [118] as shown in Figure 5(d).

**Figure 6:**
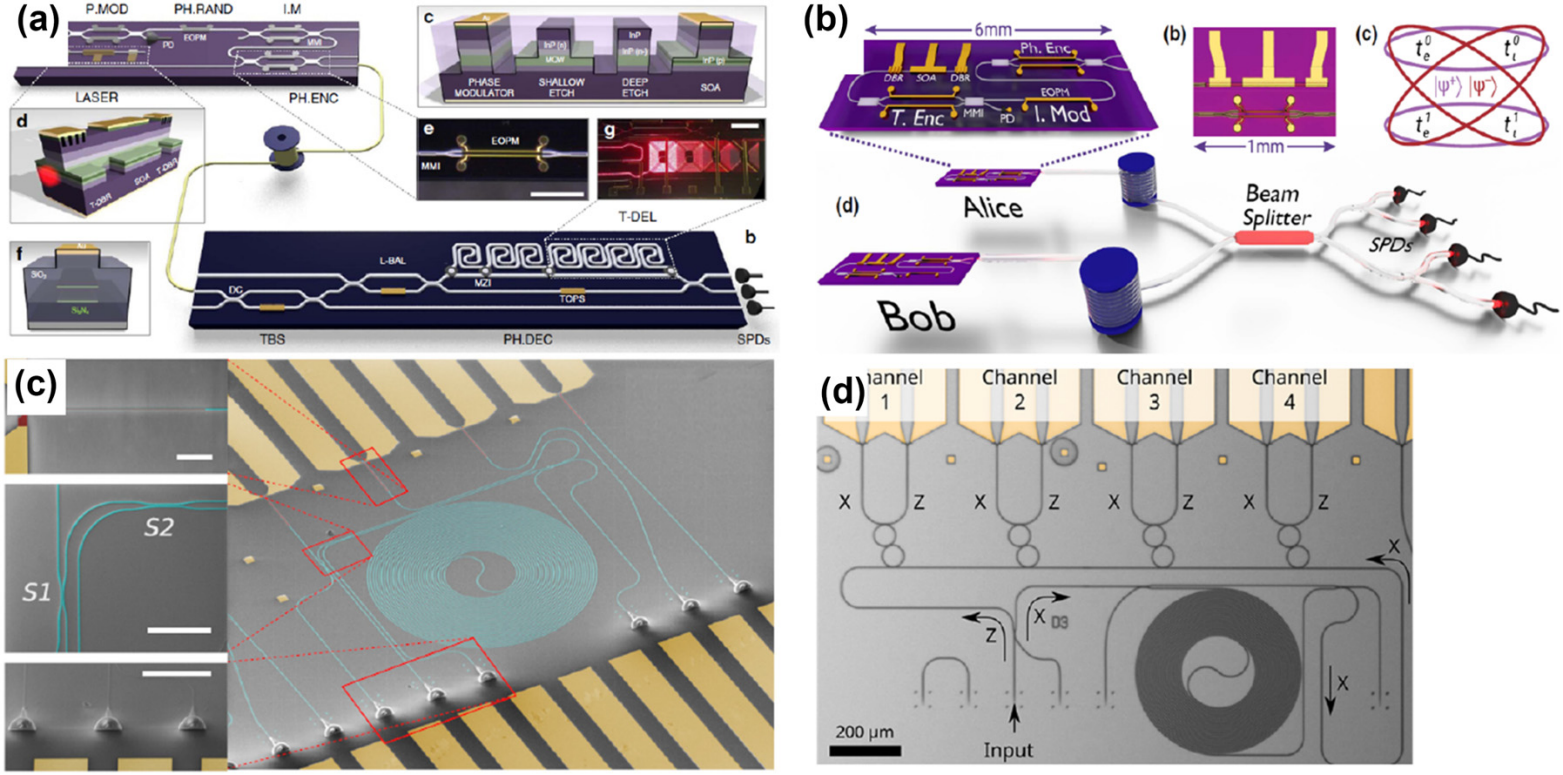
Different integrated QKD systems: (a) integrated photonic devices for multiprotocol QKD using InP transmitter, continuously tunable laser diode, photodiode, and multimode interferometers [131]; (b) integrated MDI-QKD using on-chip DBR laser, MZI elements, and nanowire single-photon detectors. Reprinted with permission from Semenenko et al. [132] © The Optical Society. (c) SEM image of QKD receiver chip with optical coupler, waveguide, splitter, optical delay lines, and nanowire single-photon detectors [133], and (d) SEM image of a four-channel time-bin QKD receiver chip. Reprinted with permission from Beutel et al. [134] © The Optical Society.

### 5.3 On-chip quantum communication

Unconditional secure information exchange is a demanding requirement for both governments and individuals. Over the past few decades, quantum key distribution (QKD) with security fundamentally guaranteed by the laws of physics has grown rapidly from lab demonstrations to the deployment of commercially available systems connecting distant cities. Demonstrated by the recently launched quantum satellite, space-to-ground QKD has linked locations over 1200 km apart [135], [136], [137], and even intercontinental quantum communication over 7600 km [138] has been realized. In general, a QKD system includes a signal-sending part for generating required photon states and a signal-receiving part for photon state detection. Integration efforts have been made to lower the size, cost, and energy consumption of both ends. Ideally, a “sender chip” using integrated light sources with polarization modulators, phase modulators, and power attenuators could efficiently generate certain photon states (e.g. BB84 with polarization or phase encoding), while a “receiver chip” integrated with many single-photon detectors, optical circuits, and control electronics could register the signal photons to decode the information [132, 139]. There have been several experimental demonstrations of silicon photonic transmitters for polarization [140], time-bins [131, 141] and space division multiplexing encoding [142]. An intercity metropolitan QKD test was performed using a silicon photonics encoder, reaching a quantum communication distance over 42 km [143]. Another recent experiment using silicon photonics realized the chip-based transmitter and receiver for continuous-variable QKD [144]. In a recent demonstration, a four-channel silicon nitride-based integrated QKD receiver achieved a total secret-key rate of up to 12.17 Mbit/s at a 3.35 GHz clock rate using wavelength-division de-multiplexing and waveguide-integrated superconducting nanowire single-photon detectors [134]. Besides QKD, chip-based quantum teleportation has also been demonstrated using an integrated photonics platform [145]. For more detailed QKD-related protocols, implementation, security analysis, and attacking risks, the reader can refer to [146, 147]. A overview of integrated QKD chips is shown in Figure 6.

### 5.4 Optical neural networks for machine learning

In the era of big data, artificial intelligence has greatly revolutionized the modern world and has applications in many areas, for example, image and language analysis, self-driving vehicles, and the famous alpha Go [148]. Currently, electronic circuits are still the prevailing computing power support for artificial intelligence, especially promoted by GPU calculations, however, the Von Neumann architecture cannot meet the increasing demand for ultra-large-scale information processing, limited by energy consumption and electronic interference [149, 150]. Light, as an excellent information carrier, which travels with fast speed and high parallelism, can solve electronic defects and the research of optical neural network (ONN) can boost the development of artificial intelligence with energy and time efficiency [151, 152].

**Figure 7:**
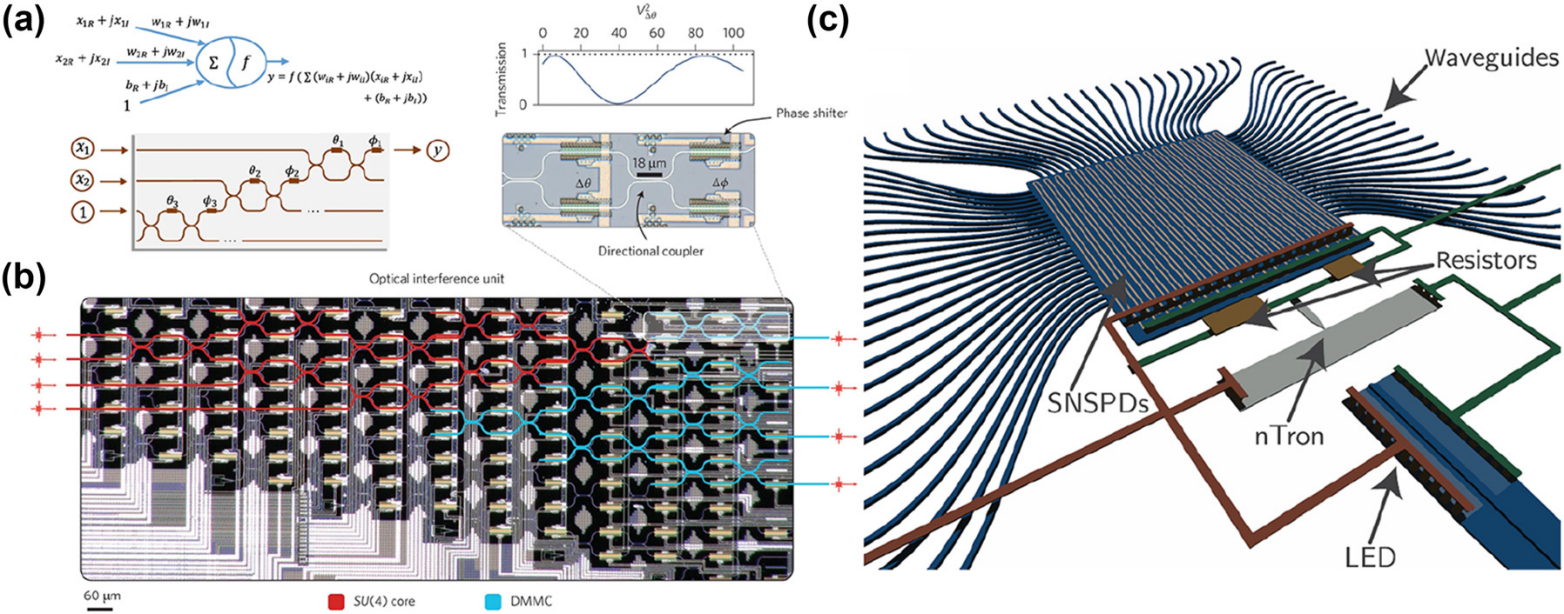
Optical neural networks with integrated photonic devices: (a) schematic of a complex-valued neuron and its implementation based on integrated MZIs [153]. (b) A micrograph illustration of an optical neural network that can perform both matrix multiplication (red circuit) and attenuation (blue circuit). The enlarged figure is an example of MZI which tunes the internal phase [154]. (c) Schematic overview of a stingray neuron using SNSPDs and LED, which is compatible with large-scale networks. Reprinted figure with permission from Shainline et al. [155], Copyright 2017 by the American Physical Society.

The optical implementation of neural networks basically contains two parts: linear operation and nonlinear activation, which can be seen as linear multiplication and summation operations, as shown in Figure 7(a), where a complex-valued neuron is implemented by a mesh of MZI [153]. In a fully connected linear network, each neuron in the output layer is a weighted sum of all input neurons — which can be mathematically represented as a matrix-vector multiplication. Such multiply-accumulate operations can be experimentally implemented by meshes of Mach–Zehnder interferometers (MZIs), as in reference [154] and Figure 7(b). The central idea is to use the principle of interference to implement linear operations, with the tunability of phase shifters in MZIs, the ONN can implement any operation on the input states. Normally the demanding resources (such as MZIs) for a *N* dimension input is *N*^2^, recently, space-efficient integrated diffractive cells are demonstrated [156] to further reduce footprint and energy consumption. Nowadays, on-chip ONNs have been extensively realized for the prediction of molecular properties [157], graph representation learning [158], noise-resilient learning [159], bacterial foraging training [160], and image classification [161]. In recent research, *in situ* training of ONNs was realized by a fully-integrated coherent optical neural network, including integrated coherent transmitter, matrix multiplication unit, nonlinear function unit and on-chip detection [162]. To push the physical limit of energy efficiency, a novel spiking neural network was recently demonstrated to perform neuromorphic computing [155, 163] using the combination of integrated photonics and SNSPDs, as shown in Figure 7(c). Such a device has the potential to perform 10 times more operations compared to the human brain with much less energy cost. Another great advance in the field of optical neural networks is the emergence of quantum machine learning [164, 165]. With unique quantum features like superposition and entanglement, quantum machine learning algorithms can outperform their classical counterpart with faster speed and fewer resources [166], [167], [168], [169], [170].

### 5.5 Integrated quantum Lidar system

Light detection and ranging, known as “Lidar”, is a powerful technology for environmental monitoring, remote target recognition, forest mapping on the earth’s surface, and sea fog measurements on ocean [171], [172], [173], [174]. It detects scattered or reflected light to acquire distance or depth information of remote targets. Typically, a Lidar system consists of pulse laser sources, beam splitters, transceivers, time-correlated single-photon counting electronics, and photodetectors. With the increase in measurement distance, after tens of kilometers, only a few photons can travel back to the detection end, thus the use of single photon detectors can efficiently improve Lidar systems’ detection range, depth accuracy, and acquisition time. The superconducting nanowire single-photon detectors developed in the past decades with high efficiency, low timing jitter, and dark count rates [17] are becoming the popular choice for recent Lidar systems. A comprehensive review of SNSPD-based Lidars can be found in [175]. In the coming future, photon number resolving detectors and efficient mid-infrared detectors will open new detection windows and capabilities for Lidar systems [176]. Also, with the development of high-power on-chip laser, photonic integrated circuits, and detector technology, monolithic Lidar chips would enable more compact, space-compatible Lidar applications in the future [177, 178].

### 5.6 Meta-surface for integrated quantum optics circuits

**Figure 8:**
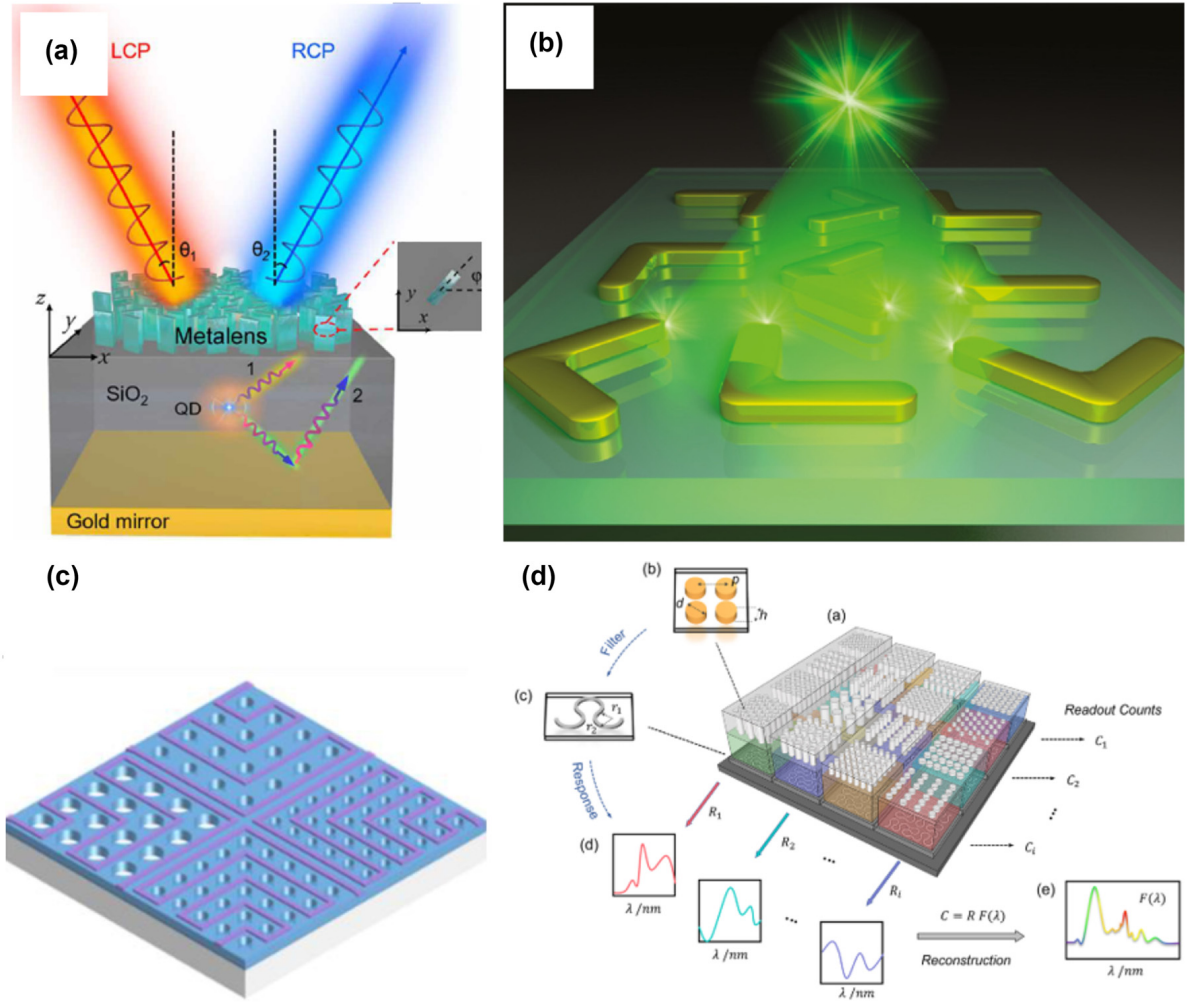
Integration of metasurface with quantum emitters or quantum detectors. (a) Manipulating QD emission using metasurface to achieve on-demand spin state control [184]; (b) illustration of a single quantum emitter interacting with metasurface for purcell enhancement [185]; (c) single-photon spectrometer using metasurface array with superconducting nanowire deployed in the region between periodic holes [186], and (d) computational spectrometer consisting of a 4 × 4 superconducting nanowire arrays and 3D-printed metasurface [187]. Reprinted with permission from Xiao et al. [187], Copyright 2022 American Chemical Society.

With the improvement of high-precision nanofabrication, recent years have seen great progress and increased interest in the field of metasurfaces, which typically contain periodic sub-wavelength metallic/dielectric structures that resonantly couple to the electric and magnetic fields of the light wave [179, 180]. Metasurfaces offer unique solutions to realize unconventional phenomena, for example, negative refraction, achromatic focusing, and electromagnetic cloaking [181], [182], [183]. The applications of metasurfaces have also been extended from traditional optics to quantum optics, where single photons sources, entangled photons, and single-photon detection are fundamentally required. For the quantum dots emitters described in Section 2, the random photon emission issue compromises their use and especially hinders the on-demand manipulation of their spin states. As shown in [184] integrating QDs with metasurface leads to on-demand generation and separation of the spin states of the emitted single photons along any arbitrary engineered direction. Also, Purcell enhancement can be realized using metasurface with QDs [185]. When combining metasurface with superconducting nanowire single-photon detectors as described in Section 3, the functionality of these quantum detectors can be greatly extended, for example, achieving spectrum reconstruction on chip [186, 187]. In the future, combining metasurface with quantum optics elements on-chip will offer new possibilities for controlling single-photon emission, single-photon state manipulation, and single-photon detection and imaging. For more detailed integrated metasurface applications in quantum optics, we refer to [188]. Representative works of meta-surface integrated emitters and detectors are shown in Figure 8.

## 6 Outlooks on future quantum photonics technologies

After decades of development, integrated (quantum) photonics—the science and technology of generating, controlling, and detecting photons on a chip scale-has benefited different industries and society. For example, in telecommunications, where bandwidth and security are greatly demanded and photonic integrated circuits (PICs) offer a viable solution; Other emerging application areas, including quantum photonic computing, bio-photonics sensing, environmental monitoring, and disease diagnosis are also witnessing game-changing breakthroughs triggered by the rapid development of quantum integrated photonic technology. Here, we present two promising envisioned photonic circuit experiments that could further boost the impact of nanowire-based integrated photonics in science and technology.

### 6.1 On-chip quantum information processing

**Figure 9:**
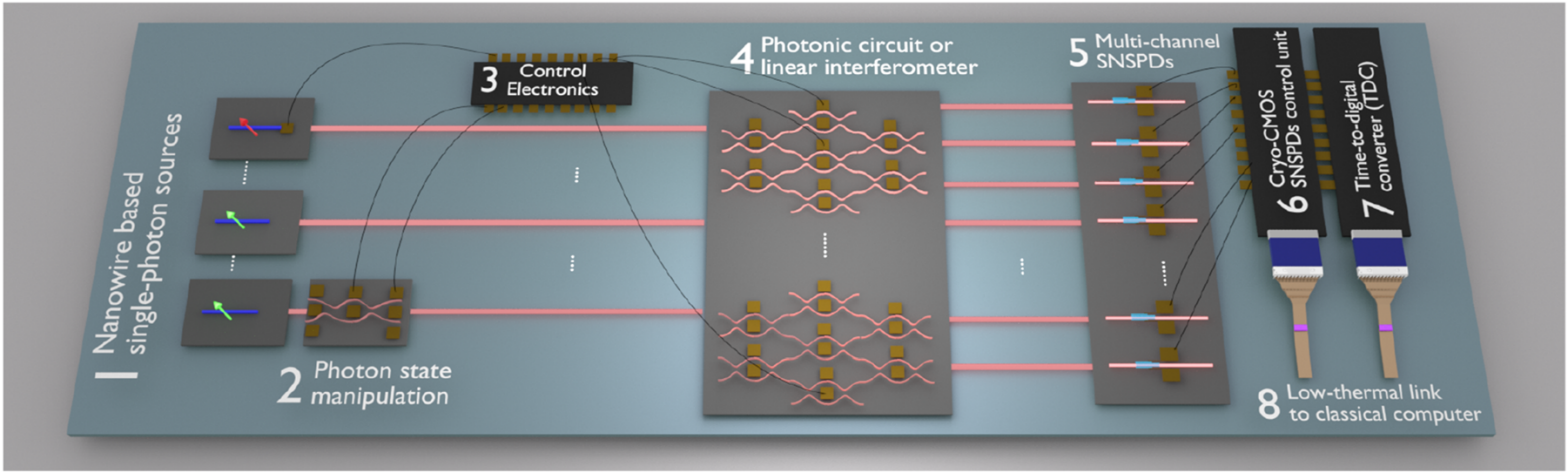
Illustration of an integrated nanowire-based photonic chip to be developed in the future. In this figure, optically excited nanowire QDs sources are shown in green arrows while the electrically pumped single-photon source is shown in red arrows; optical connections between different components are highlighted in pink lines, and electrical contacts are represented by orange squares; waveguide-integrated superconducting nanowire single-photon detectors are marked by cyan wires, and integrated control electronic circuits, for example, cryo-CMOS or TDCs, are shown in black rectangles with multiple bonding pads.

Recently, the design and production of integrated photonics started to merge into the mainstream of the electronic industry. Such hybrid chips [7] take the complementary advantages of both platforms to perform sophisticated tasks. As illustrated in Figure 9, the hybrid photonic/electronic integrated circuit contains optically pumped (green) and electrically driven (red) nanowire QDs as single-photon sources. After emission, the photon states can be tuned by additional on-chip elements (e.g. phase shifters [189]). Multiple photons with precisely controlled initial states are then ejected into the linear interferometer to interact with different photonic quantum computing or simulation protocols. Afterward, the output results (photons) are registered by multi-channels on-chip SNSPDs with integrated control CMOS circuits and time-to-digital (TDC) converters. Such a hybrid chip can be mounted in a compact cryostat without using many coaxial cables, which helps to improve system scalability and reduce the total heat load. In the future, to realize mass production of such proposed hybrid chips, each containing millions of elements, electronics, CMOS-compatible optics, and dedicated superconductor fabrication are simultaneously needed at a foundry level. Also, automatic pre-testing equipment of such chips is significantly important to be developed (e.g. cryogenic probe station [190]), where artificial intelligence algorithms can also help to improve failure analysis [191].

### 6.2 On-chip quantum bio-sensing

Different classes of single-photon emitters have been employed for bio-photonic imaging and spectroscopy. Each of these emitters has its own advantages and weaknesses. To this end, quantum dots were among the first emitters to be explored and have already come a long way. Most commonly in biomedical applications and fluorescence microscopy, quantum dots (and in general single-photon emitters) are utilized as biomarkers [192], [193], [194], [195], [196] in which their position (localization), color, brightness, lifetime, etc. is linked to a certain biological/chemical factor. Among the important remaining challenges ahead of bio-quantum sensing with quantum dots are chemical toxicity and optical attenuation, i.e. the light from the emitter is highly attenuated by the tissue before reaching the detection optics. Infrared quantum dots can benefit from enhanced transparency of the tissue. Synthesis of high-quality infrared emitting quantum dots [197, 198] as well as efficient and precise detection of those photons [199, 200] have achieved promising results but require further progress. High quantum yield emitters as well as sensitive and low noise detectors with a large active area can further boost the impact of infrared bio-imaging.

As described in the previous sections, integrated photonic circuits can generate, transmit, and detect a broad band of optical signals on-chip. This naturally offers the ability to simultaneously detect and identify different biological objects (e.g. single-virus [201], proteins [202], and single-molecule [203]), which is one of the key requirements for disease diagnostic and drug developments.

**Figure 10:**
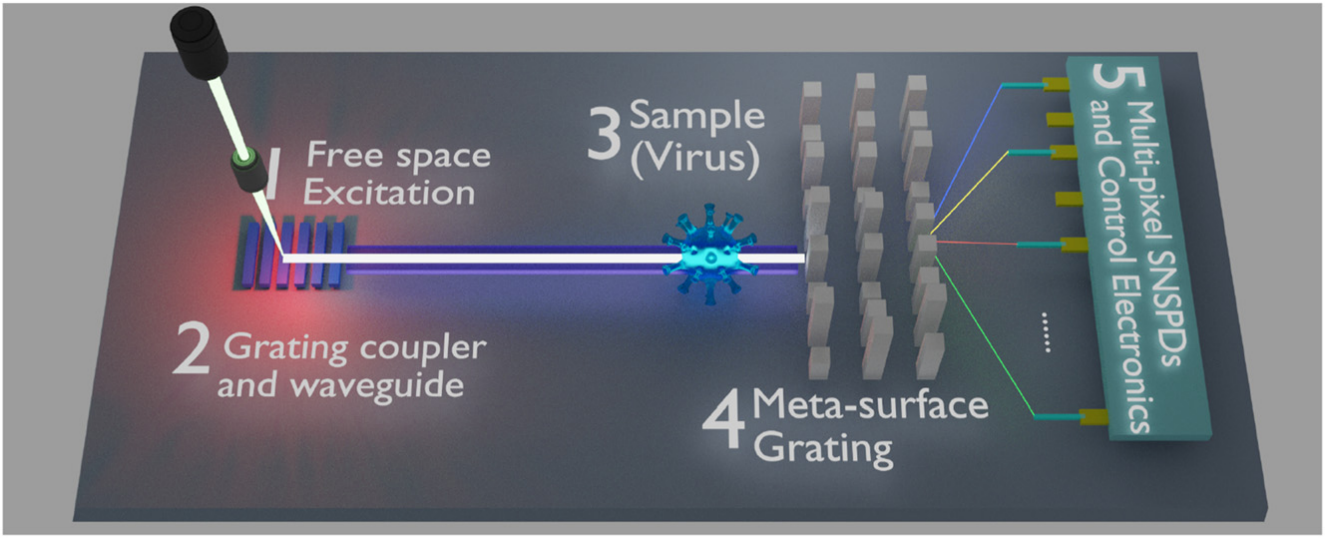
A proposed integrated quantum sensing chip for single-virus testing, including free space excitation laser, grating couplers, optical waveguide, the sample under test (single-virus as an example, can also be a single-molecule or protein, etc.), meta-surface grating, and multi-pixel SNSPDs.

Integrated photonic circuits can help to create such a highly sensitive, multi-functional platform on a chip scale. Also, by using single-photon detectors, the sensitivity of such systems can be improved to reach their quantum limits. As depicted in Figure 10, a broadband excitation light signal transmits from free space to the chip with the help of efficient grating couplers. The waveguide delivers light to the samples under test (a virus in this case). Afterward, the transmitted or scattered photon signal pass through the meta-surface grating to acquire spectral information with multi-pixel SNSPDs at the detection ports. With precise spectral information, the identity or structure of bio-samples can be efficiently acquired. Such chips hold great potential in both scientific labs and biochemical industries, and again, nanowire-based devices are the key enabling elements in the proposed systems.

## 7 Conclusion and future perspectives

With the 2022 Nobel physics prize awarded to the scientists who opened the quantum optics field using single-photons and entangled photon pairs, the field of quantum photonics science is expected to gain more attention. After decades of development, this field has witnessed great developments in both fundamental theory and real-world applications. The applications of nanowires QDs and SNSPDs are extending from proof-of-principle demonstrations to large-scale quantum computing, quantum simulation, and quantum sensing. Integrated and hybrid quantum photonic solutions are promising approaches for developing next-generation quantum hardware, where the advantages of photonics, electronics, and condensed matter physics can be combined for game-changing innovations. Looking into the future, there are still important challenges to be addressed:

– From quantum emitters’ perspective, to interface the flying qubits with the current optical fiber network, quantum emitters must be developed that operate at the telecommunication wavelength around 1550 nm. A further benefit of this advancement is enabling hybrid integration to silicon on insulator photonics, the most developed photonic platform for both classical and quantum applications. Additionally, one key objective is to interface several nanowire QDs, so that they may be used as solid-state quantum memories, and each may function as a quantum node for communication purposes. Such target application would require Fourier-limited emission and more stringent control over the emission wavelength spread in nanowire quantum dot samples. Lastly, higher photon indistinguishability without time-gating or post-processing is key for several applications highlighted in this review.
– On the detection side, significant advancements have recently been made, allowing SNSPDs to be used in new fields including biological imaging. Following this advancement, it may be advantageous to realize SNSPDs at longer wavelength ranges. This should be done in collaboration with researchers developing biological markers to maximize the detectors’ qualities in the desired wavelength range. Additionally, even though large-scale SNSPD arrays have made significant progress, additional work is still required to realize SNDPD 2D cameras with individual pixel-readout circuitry solutions. In the realms of optical imaging, sensing, and biology, this will be a game-changer. Also, a better understanding of how the superconducting films’ material properties influence their photon detection performance will help to improve SNSPDs’ yield, and ultimately bring down the cost of this technology. This will advance the commercialization of SNSPDs and expand their use to as-yet-untapped markets and research areas.

To conclude, through a systematic review of the theory, material platform, and fabrication process of the nanowires, this paper serves as a solid reference for both young and senior researchers in the integrated quantum photonics field. Additionally, we propose promising quantum photonics architectures and sensing experiments and aim at attracting a wider range of readers and invoking more collaboration between researchers in different fields.
